# The misleading label of atypical femur fracture: a call for diagnostic clarity amid biological diversity

**DOI:** 10.2340/17453674.2025.44329

**Published:** 2025-07-25

**Authors:** Jörg SCHILCHER

**Affiliations:** Department of Clinical and Experimental Medicine, Division of Orthopaedics, Linköping University, Linköping, Sweden

The characterization of atypical femur fractures (AFFs) has significantly impacted the management of osteoporosis. The term “atypical” was initially introduced to distinguish these diaphyseal fractures from the more “typical” fragility fractures based on their “atypical” anatomical location in the femoral shaft compared with the metaphyseal fracture location of most fragility fractures [1]. Despite our increasing understanding of AFFs as drug-related stress (insufficiency) fractures, the term AFF is now used for a variety of fractures with different underlying pathologies, which has led to diagnostic pluralism. Defining AFFs as antiresorptive-associated insufficiency fractures based on the well-described underlying pathophysiological mechanisms and using stringent radiographic criteria to define them could help differentiate these fractures from other types of stress fractures (fatigue and insufficiency), providing greater clarity for clinicians and researchers.

## Consequences of the present diagnostic pluralism

Despite explicit recommendations in the 2014 task force report to exclude bone metabolic disorders from a diagnosis of AFF, insufficiency fractures associated with a variety of distinct clinical entities, such as monogenic or metabolic bone disorders (e.g., osteogenesis imperfecta, hypophosphatasia, adynamic bone disease, osteopetrosis), are often classified as AFFs [2,3]. Even the classic fatigue-type stress fractures in healthy bone can be categorized under the AFF label when following the American Society for Bone and Mineral Research (ASBMR) case definition. This diagnostic convergence has significant consequences. Misclassification skews risk estimates in epidemiological studies, complicates treatment guidelines, and undermines clinician confidence in managing antiresorptive therapy.

## Current definition of AFF

AFFs are currently defined as insufficiency fractures. Insufficiency fractures develop in bone with inadequate material properties to withstand the strains of daily activities (Table 1). Fatigue fractures, conversely, occur when healthy bone is subjected to repetitive submaximal loading. Both insufficiency fractures and fatigue fractures fall under the category of stress fractures and can be associated with bone diseases, medication treatments, prolonged marches in military service, high-impact sports activities, or a combination of these.

**Table 1:** Key differences between insufficiency and fatigue type stress fractures

Feature	Insufficiency stress fracture	Fatigue stress fracture
Definition	Fracture due to *normal stress* on *altered bone*	Fracture due to *abnormal stress* on *normal bone*
Bone quality	Decreased (e.g., reduced remodeling capacity, microdamage accumulation, hypermineralized bone matrix)	Normal
Typical population	Those with altered bone tissue properties	Young, healthy individuals (e.g., athletes, military)
Mechanism	Everyday activities	Repetitive submaximal load
Radiographic findings	Peri- or endosteal reaction (callus formation), transverse fracture line formation. (Both usually occur at a later stage)	Peri- or endosteal reaction (callus), transverse fracture line

In an effort to standardize the diagnosis of AFF, the ASBMR task force issued consensus reports in 2010 and 2014. These reports clearly establish that AFF is closely associated with antiresorptive treatment and shows a characteristic clinical and radiographic profile of insufficiency fractures [4]. The ASBMR task force also outlined 5 major diagnostic criteria (Table 2) [5].

**Table 2:** Radiographic stress fracture criteria in relation to 2014 ASBMR major criteria for AFF: 4 of 5 criteria must be fulfilled to define AFF

ASBMR major criteria of AFF	Radiographic stress fracture criteria
The fracture line originates at the lateral cortex and is substantially transverse in orientation, although it may become oblique as it progresses medially	Yes
Localized periosteal or endosteal thickening of the lateral cortex (callus reaction)	Yes
Complete fractures extend through both cortices and may be associated with a medial spike; incomplete fractures involve only the lateral cortex	No
The fracture is noncomminuted or minimally comminuted	No
The fracture is associated with minimal or no trauma (e.g., fall from standing height or less)	No

However, these criteria have led to the unexpected consequence of diagnostic pluralism:

1. Using the ASBMR major criteria, fractures that are not stress fractures can be correctly defined as AFF.
2. Typical stress fractures (fatigue and insufficiency) are now called AFF.

This current confusion is further complicated by the requirement of meeting 4 out of 5 major criteria.

## ASBMR criteria, the 4 out of 5 principle

The ASBMR recommendation that at least 4 of the 5 major criteria must be present has resulted in considerable variability in clinical interpretation (see Table 2) [6]. It is essential in this context to note that only 2 of the criteria—transverse fracture line and focal cortical thickening (the callus reaction during stress fracture formation)—are radiographically specific for stress fracture morphology (see Table 2). The other 3 criteria can appear in any other type of fracture.

This means that even when the ASBMR major criteria are used correctly, there are 6 combinations of the major criteria that satisfy the AFF definition: 5 combinations where any 4 criteria are met, and one combination where all 5 are fulfilled. This can be expressed as the sum of 2 binomial coefficients:

\Binom (5)(4) + \binom (5)(5) = 5 + 1 = 6

Consequently, fractures that lack either of the 2 core radiographic features of a stress fracture (transverse fracture line and focal cortical thickening) can still meet the ASBMR definition. This undermines the very foundation of AFF as a stress fracture and blurs the distinction between antiresorptive-related metabolic changes leading to an insufficiency fracture and other significantly more common types of fragility fractures of the femoral shaft.

## Redefinition: antiresorptive-associated femoral insufficiency fracture

A redefinition following a mechanism-based framework is overdue. Considering the underlying pathophysiology, the term “antiresorptive-associated femoral insufficiency fracture” more accurately captures the nature of these lesions—insufficiency-type stress fractures that occur under normal physiological loading in the setting of antiresorptive therapy [6].

The underlying mechanism is the suppression of targeted bone remodeling, the bone’s intrinsic repair system, which has been well described in previous literature [6,7]. A central feature is the accumulation of microdamage that normally occurs during physiological loading (more extensively in the aging skeleton) but is not repaired by the targeted remodeling process because of the inhibitory effect of the antiresorptive effect on the osteoclast [8] (Figure). Additional contributory factors include altered strain distribution along the femur and changes in bone material properties.

**Figure:**
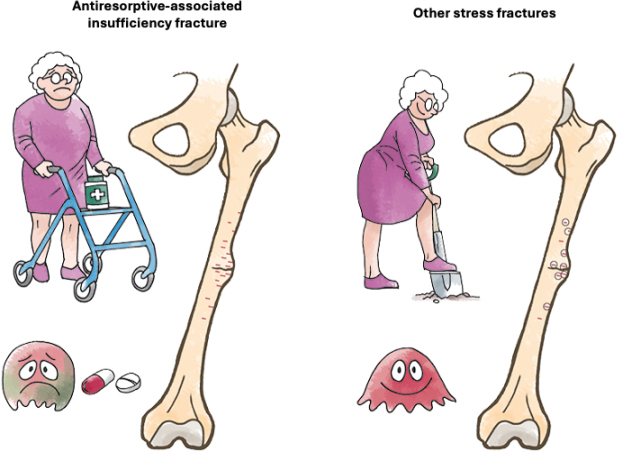
Schematic illustration of the pathophysiological differences between antiresorptive-associated stress fractures (left) and other stress fractures (right). In antiresorptive-treated bone, remodeling is suppressed due to osteoclast inhibition, leading to the accumulation of microcracks (red). In contrast, in other stress fractures, remodeling remains intact and allows for resorption of microcracks and replacement with new bone tissue (remodeling: circles around the cracks).

This reframing would clearly distinguish antiresorptive-associated femoral insufficiency fractures from stress fractures of fatigue type or metabolic origin and re-establishes it as a discrete clinical entity, consistent with the original intent. A slight adjustment of the original abbreviation would be required to fit the framework—AAFF.

## Other insufficiency types of stress fractures

The study by Zdolsek et al. [9] provides a clinical rationale for this distinction. In a comparison of patients with incomplete stress fractures, both with and without bisphosphonate (BP) exposure, BP-naїve patients with stress fractures demonstrated metabolic bone disease, pathologic femoral geometry, or a history of mechanical overload, as well as radiographic patterns indicative of ongoing remodeling, such as bone resorption at the fracture site [9]. These observations suggest that stress fractures in BP-naїve individuals maintain remodeling potential at the stress lesion (Figure), similar to fatigue fractures seen in young athletes [10]. Histologically, BP-treated patients exhibited a higher bone volume fraction (BV/TV) and the presence of giant osteoclasts, a sign consistent with impaired remodeling and suppressed turnover. These findings reinforce the observation that antiresorptive-associated insufficiency fractures constitute a unique phenomenon that needs to be distinguished from other entities.

## Conclusion

Revisiting the nomenclature of AFF within a mechanism-based framework is both scientifically justified, clinically necessary, and makes the enigmatic fracture entity of AFF superfluous. As our understanding of the pathophysiology and epidemiology has evolved over the last 2 decades, these fractures should be recognized and referred to as treatment-associated insufficiency fractures characterized by the distinctive radiographic features of stress fractures. To provide objective and specific assessments in both clinical and research settings, the radiographic criteria for stress fractures—namely, a transverse fracture line and focal cortical thickening (callus reaction)—should be mandatory for defining complete fractures, while the transverse fracture line may be absent in incomplete fractures. This shift toward a mechanism-based nomenclature would enable research efforts to more accurately quantify the excess risk of antiresorptive-associated femoral insufficiency fractures compared with other types of insufficiency, fatigue, or fragility fractures within the population and add greater clarity for clinicians and researchers.

## Notes

Complete disclosure of interest form according to ICMJE is available on the article page, doi: 10.2340/17453674.2025.44329

Handling co-editor: Keijo Mäkelä

*Acta* thanks Hannu T Aro for help with peer review of this manuscript.
